# FLVCR1 Predicts Poor Prognosis and Promotes Malignant Phenotype in Esophageal Squamous Cell Carcinoma *via* Upregulating CSE1L

**DOI:** 10.3389/fonc.2021.660955

**Published:** 2021-03-25

**Authors:** Suna Zhou, Mingxin Zhang, Chao Zhou, Yinnan Meng, Haihua Yang, Wenguang Ye

**Affiliations:** ^1^ Laboratory of Cellular and Molecular Radiation Oncology, The Affiliated Taizhou Hospital, Wenzhou Medical University, Taizhou, China; ^2^ Department of Radiation Oncology, The Affiliated Taizhou Hospital, Wenzhou Medical University, Taizhou, China; ^3^ Key Laboratory of Minimally Invasive Techniques & Rapid Rehabilitation of Digestive System Tumor of Zhejiang Province, Taizhou, China; ^4^ Department of Gastroenterology, The First Affiliated Hospital of Xi’an Medical University, Xi’an, China; ^5^ Department of Gastroenterology, The Affiliated Taizhou Hospital, Wenzhou Medical University, Taizhou, China

**Keywords:** FLVCR1, ESCC, CSE1L, migration, growth

## Abstract

**Objective:**

Dysregulation of feline leukemia virus subgroup C receptor 1(FLVCR1) expression has been investigated in several tumors. However, the expression and role of FLVCR1 in esophageal squamous cell carcinoma (ESCC) remain largely unknown.

**Methods:**

FLVCR1 expression in tissues was measured by immunohistochemical staining (IHC). Celigo assay, MTT assay, colony formation, caspase 3/7 activity analysis, wound healing assay, Transwell migration, and invasion assay were applied to assess the effects of FLVCR1 on ESCC tumorigenesis. Coimmunoprecipitation (Co-IP) and liquid chromatography-mass spectrometry (LC-MS) were used to identify protein interactions with FLVCR1. An *in vivo* imaging system (IVIS) was used to investigate the functions of FLVCR1 on the growth and metastatic capability of ESCC cells in a xenograft model and a tail vein metastasis model.

**Results:**

Elevated expression of FLVCR1 was detected in ESCC tissues and predicted poor survival. Upregulated FLVCR1 was positively correlated with lymph node metastasis (N stage) and late tumor-node-metastasis (TNM) stage. FLVCR1 knockdown inhibited cell proliferation and colony formation ability, induced cell apoptosis, and repressed cell migration and invasion of ESCC *in vitro*. Inhibition of FLVCR1 markedly repressed tumorigenicity and metastasis of ESCC cells *in vivo*. Mechanistically, chromosome segregation 1–like (CSE1L) was identified to interact with FLVCR1 using a Co-IP assay. Moreover, the inhibitory effect of FLVCR1 knockdown on proliferation and migration was counteracted by the exogenous expression of CSE1L.

**Conclusion:**

FLVCR1 plays a pivotal role in ESCC cell survival, growth, and migration. These functions may be partially dependent upon the protein interaction between FLVCR1 and CSE1L. In addition, FLVCR1 can be applied as a clinical prognostic marker for patients with ESCC.

## Introduction

Esophageal squamous cell carcinoma (ESCC), the predominant type of esophageal carcinoma in Asia, is one of the most aggressive tumors with high incidence and mortality (1, 2). China is one of the countries with the highest incidences of esophageal carcinoma worldwide (3). Although the morbidity and mortality rates of ESCC are decreasing in China, the number of new cases and deaths accounts for more than 50% of the world’s data (3). Despite improvements in both diagnostic and therapeutic technology, the 5-year survival rate of patients with ESCC remains proximately 20.9% in China (4). High metastatic ability and tumor recurrence are the leading causes of mortality in ESCC patients with or without surgery (5). The occurrence and development of ESCC is a complex process that involves multiple steps and genes. Thus, a better understanding of the role and the molecular mechanisms involved in the carcinogenesis of ESCC is expected to improve early diagnosis and effective targeting treatment.

FLVCR1, containing 12 hydrophobic transmembrane domains, is a member of the major facilitator superfamily and can transfer small solute molecules (6). FLVCR1 encodes two heme exporters, FLVCR1α localizing on the plasma membrane and FLVCR1β restricted to the mitochondria (6). The abnormal level of heme is essential for tumor progression and metastasis (7, 8). Chiabrando et al. reported that the heme exporter FLVCR1 is crucial for the survival of neuroblastoma cells by regulating heme metabolism (9). Peng et al. found that FLVCR1 plays a critical role in promoting the tumorigenicity and proliferation of synovial sarcoma *via* impeding apoptosis and autophagy (10). The overexpression of FLVCR1 in hepatocellular carcinoma is associated with higher disease staging, vascular invasion, histologic grade, and poorer outcomes (11). In summary, these results suggest that FLVCR1 may function as an oncoprotein during the process of tumor development. However, the role and molecular mechanisms of FLVCR1 involved in the malignant transformation of ESCC are still unknown.

In the present study, high expression of FLVCR1 was also detected in ESCC and played essential roles in promoting proliferation, invasion, and metastasis of ESCC and served as a poor prognostic marker. The results of co-immunoprecipitation (Co-IP) combined with western blotting suggested that CSE1L bound to the FLVCR1 protein. Further investigation showed that CSE1L was indeed involved in FLVCR1-mediated modulation of proliferation and migration of ESCC. Our study reveals that FLVCR1 may be a potential therapeutic target for ESCC treatment.

## Materials and Methods

### Clinical Samples

Two sets of ESCC samples were included in this study. The first set containing 31 matched pairs of ESCC tumor tissues and normal tissues was purchased from Shanghai Outdo Biotech Inc (OD-CT-DgEso03-002) and used to analyze FLVCR1 expression at the protein level. FLVCR1 expression and the correlation between FLVCR1 expression and clinicopathological features were further analyzed in the second set containing 103 ESCC samples with clinical follow-up information. The second set of ESCC samples was taken from patients who underwent surgery at The Affiliated Taizhou Hospital of Wenzhou Medical University from January 2006 to December 2008. The follow-up term is from 6.6 to 9.5 years. The last follow-up time was July 2015. The Institutional Review Board of Taizhou Hospital approved the study, and informed consent was obtained from each patient.

### Cell Lines

The human ESCC cell lines TE-1, KYSE-150, Eca-109, and Ec-9706 and the human normal esophageal epithelial cell line HEEC used in this study were purchased from Shanghai Cell Biology Institute of Chinese Academy of Sciences (Shanghai, China). Cells were maintained in RPMI 1640 supplemented with 10% FBS (Gibco, Grand Island, NY) and penicillin (100 IU/ml)/streptomycin (100 μg/mL) (HyClone, Logan, UT, USA) at 37°C with 5% CO2.

### Immunohistochemical Staining

Briefly, immunohistochemical staining (IHC) was performed as follows. The tissue sections were deparaffinized in xylene, rehydrated in graded ethanol and soaked in distilled water. After heat-mediated citric acid antigen retrieval, the tissue sections were incubated with the primary antibody for FLVCR1(Abcam, ab70838, rabbit polyclonal antibody) at a dilution of 1/500 at 4°C overnight and incubated with horseradish peroxidase (HRP)-conjugated antibody (Thermo Scientific, USA) for 60 min at room temperature. Next, the solutions were replaced with DAB solutions (Gene Tech, Shanghai, China). Finally, these processed tissue sections were observed under a microscope and then counterstained with hematoxylin. Known immunostaining positive slides served as a positive control. Slides incubated with an irrelevant rabbit antiserum were used as a negative control. The expression of FLVCR1 was scored according to the percentage and intensity of positively stained cells. As shown in **Supplementary 1**, the percentage of positive cells indicated the following scores: negative scored 0, 1-25% scored 1, 26-50% scored 2, 51-75% scored 3, and 76-100% scored 4; the intensity of positive cells indicated the following scores: negative scored 0; I grade scored 1; II grade scored 2; and III grade scored 3. The product of these two scores was defined as the total score. In this study, patients with a score≤ 6 were designated as the low-expression group, while patients with a score > 6 were designated as the high-expression group. The expression of FLVCR1 was assessed in a blinded manner by two observers.

### Generation of Stable Overexpression or Downregulation Cell Lines

To downregulate the expression of FLVCR1, the most effective siRNA sequences were subcloned into the GV115(hU6-MCS-CMV-EGFP) vector to generate an shFLVCR1 recombinant lentivirus (GeneChem Corporation, Shanghai, China). TE-1 and KYSE-150 cells were infected with the recombinant lentivirus. To upregulate the expression of FLVCR1 or CSE1L, the cDNA of FLVCR1 or CSE1L was amplified and subcloned into GV610(Ubi-3FLAG (Sigma)-MCS-CMV-EGFP-SV40-puro) to generate a FLVCR1-OE or CSE1L-OE recombinant lentivirus, and then KYSE-150 cells were infected with the recombinant lentivirus. Subsequently, cells were selected with puromycin (2 μg/ml) for 2 weeks.

### Quantitative Real-Time Polymerase Chain Reaction (qRT-PCR)

By using Trizol (Invitrogen (Invitrogen, Carlsbad, CA), total RNA was extracted from cells followed by reverse transcription of RNA to cDNA using the PrimeScript RT reagent Kit (Takara, Shiga, Japan). qRT-PCR was performed with SYBR Green Real-time PCR Master Mixture (Takara, Shiga, Japan) using an Mx3000Ps qRT-PCR system (Applied, Foster City, CA, USA). The relative gene expression was normalized to the internal control GAPDH using the formula 2^-ΔΔCt^. The PCR conditions were as follows: pre-denaturing at 95° C for 15s and 45 cycles at 95°C for 5s and 60°C for 30s. The primers used in study were as follows: FLVCR1: 5′- GTGAGATTGGAGGGACAA GTAT-3′(upstream), 5′- TTTCATGGATGAGGAAAACG-3′ (downstream); CSE1L: 5′- CAGAAC ACGCTGACAAGTATCT-3′(upstream), 5′-AGCCCTGCGTCTAGTATCAATA-3′ (downstream); GAPDH:5′-TGACTTCAACAGCGACACCCA-3′ (upstream), 5′- CACCCTGTT GCTGTAGCCA AA-3′(downstream).

### Western Blot Assay

Briefly, total cell protein was extracted using RIPA buffer (Beyotime, Shanghai, China) and then quantified by a BCA Protein Assay Kit (Beyotime). Protein samples were separated by 10% SDS-PAGE followed by transfer onto PVDF membranes, which were then blocked with 5% skim milk for 1 h. Subsequently, cells were incubated with anti-rabbit FLVCR1 (Abcam, ab70838, which is a rabbit polyclonal antibody, and can be used to analyze FLVCR1 protein expression in IHC-P and WB. The immunogenicity: Synthetic peptide conjugated to KLH derived from within residues 500 to the C-terminus of Human FLVCR.) or CSE1L (Abcam, ab96755) or anti-mouse Flag (Sigma, F1804) at 1/300,1/500, and 1/2000 dilutions for 12h at 4°. The anti-mouse GAPDH antibody (Santa-Cruz, sc-32233) was used as a loading control at a dilution of 1:2000. HRP-conjugated goat anti-rabbit or goat anti-mouse IgG antibody (CST, #7076, #7074) was applied as a secondary antibody at a dilution of 1:2000. The gray value of the protein band was analyzed by ImageJ software.

### Celigo Assay

After transfection, the cells were seeded into 96-well plates at a density of 2x10^3^ cells/well. The number of cells with green fluorescence was scanned daily for 5 days by a Celigo^®^ Image Cytometer (Nexcelom, Lawrence, MA, USA) from the second day after plating.

### MTT Assay

Cells were seeded in 96-well plates at a density of 2x10^3^ cells/well after transfection. 20 µl 5 mg/ml MTT (Genview, Beijing, China) was added for the last 4 h before incubation termination at 37°C.

Then, the medium was removed and 100 µl DMSO (Shanghai Shiyi Chemical Reagent, Shanghai, China) was added to dissolve the formazan crystals. After 2-3 min of oscillation, the viable cells were measured at 490 nm by a microplate reader (Tecan, Mnnedorf, Switzerland).

### Colony Formation Assay

After infection, TE-1 and KYSE-150 cells were seeded into six‐well plates at 600 and 800 cells/well followed by further incubation for 8 days, respectively. Plates were washed once with PBS, fixed with 4% paraformaldehyde (Sinopharm Chemical Reagent, Shanghai, China) for 30–60 min and washed again. Then, they were stained with crystal violet (Shanghai Biotechnology, Shanghai, China) for 10–20 min and washed with ddH2O several times. Finally, the colony number was counted and photographed under an IX71 fluorescence microscope (Olympus, Tokyo, Japan).

### Caspase 3/7 Activity Analysis

After infection, TE-1 and KYSE-150 cells were seeded at a density of 2x10^4^ cells/well into 96-well plates. Ten milliliters of Caspase-Glo 3/7 buffer and substrate (Promega, Madison, WI, USA) were blended down to Caspase−Glo reaction solution. After the preparation of a reaction solution, each well was added with 100 µl Caspase-Glo reaction solution followed by 2 h incubation at room temperature. Next, the luminescence intensity was detected by a microplate reader at 570 nm.

### Wound Healing Assay

ESCC cells were seeded into 96-well plates at a density of 5x10^4^ cells/well. FBS-free medium was replaced with 10% FBS-containing RPMI-1640 medium until the monolayer adherent cells reached 90% confluence. A wound was scraped across the cell monolayer. Photomicrographs were taken with an IX71 fluorescence microscope at zero time points and after 4 h and 8 h. A Celigo^®^ Image Cytometer was used to quantify the wound area and to calculate the ratio of migration.

### Transwell Migration and Invasion Assay

After infection, TE-1 and KYSE-150 cells were respectively seeded onto the upper chamber with or without Matrigel (Corning Costar, Cambridge, MA, USA) at 8×10^4^ and 1×10^5^ cells/well. RPMI-1640 medium containing 30% FBS was added to the lower chamber. A cotton swab was used to wipe the non-migrated/non-invasive cells after 20-48 h incubation. The migrated/invasive cells were fixed and stained with Giemsa, followed by cellular count and photograph under a microscope.

### *In Vivo* Growth and Metastasis Assay

For the *in vivo* tumor growth assays, 4×10^6^ KYSE-150 cells stably transfected with shFLVCR1 (KD) or the negative control (NC) were separately transplanted subcutaneously into the right-back flank of 4-week-old female BALB/c nude mice (Shanghai SLAC Laboratory Animal, Shanghai, China). They were divided into KD and NC groups with 10 nude mice per group. Tumor sizes were measured every other 4 days until the mice were humanely sacrificed and calculated using the formula: (length × width × width)/2. The mice were intraperitoneally injected with D-luciferin and photographed with the In Vivo Imaging System (IVIS)-Lumina LT (Perkin Elmer, Waltham, USA) at the end of the fourth week after transplantation. Then, the tumors were harvested and weighed when the mice were euthanized by CO2 asphyxia. For *in vivo* metastasis assays, 2×10^6^ KYSE-150 cells stably transfected with shFLVCR1(KD) or the negative control (NC) (10 per group) were separately injected into the lateral tail veins of 5-week-old female BALB/c nude mice. After 8 weeks, the mice were also photographed under the In Vivo Imaging System (IVIS)-Lumina LT and euthanized to harvest their lungs, livers, and tumors.

### Co-IP and LC-MS

KYSE-150 cells stably transfected with 3×Flag-tagged-FLVCR1(OE) or control (NC) were washed two times with PBS for further study when the cells reached more than 80% confluence. Total cell protein was extracted using immunoprecipitation lysis buffer (Beyotime, Shanghai, China) and then quantified by a BCA Protein Assay Kit. Equal amounts of protein from the NC and OE groups were co-immunoprecipitated with anti-FLAG beads (Sigma, A2220). The products of Co-IP were exchanged and concentrated with 3×FLAG peptide (Sigma, F4799). The concentrated samples were analyzed by SDS-PAGE utilizing Coomassie Blue staining. Protein bands were excised from the gel and digested into peptides by trypsin. The sample of each peptide was identified by the LC-MS method, and then PD/MASCOT software was used to search the protein database to identify FLVCR1-interacting proteins. Finally, these potential FLVCR1-interacting proteins were summarized in the form of a gene network diagram by using GO analysis and KEGG analysis.

### Statistical Analysis

All experiments were repeated in triplicate. The data are represented as the mean ± SD in our study. Statistical analyses were performed using SPSS software (version 23.0) and GraphPad Prism software (version 8.0). The correlation between FLVCR1 expression and clinicopathological factors of ESCC was analyzed by the Chi-square test and Fisher’s exact probability test. The prognosis of FLVCR1 was identified by using univariate and multivariate Cox regression analyses. Survival analysis was performed by the Kaplan–Meier method and the log-rank test. The comparison between the groups was analyzed *via* Student’s t-test and Mann–Whitney U test. P<0.05 was defined as statistically significant.

## Results

### FLVCR1 Is Overexpressed in ESCC and Associated With Poor Prognosis

**Figure 1:**
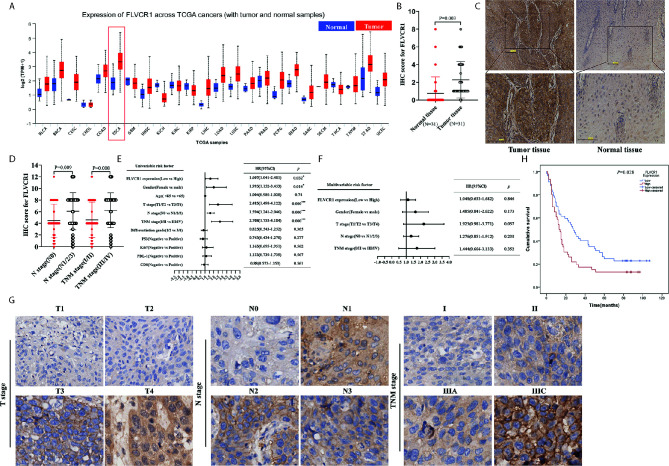
FLVCR1 expression is upregulated in ESCC tissues and predicts a poor clinical outcome. **(A)** Pan-cancer analysis of FLVCR1 based on TCGA data. **(B)** The IHC scores of FLVCR1 in 31 matched normal tissues and ESCC tissues. **(C)** Representative photographs of FLVCR1 IHC staining in normal and tumor tissues. (Scale bar: 100μm; Magnification×200.) **(D)** The IHC scores of FLVCR1 in ESCC patients with different N stages and TNM stages. **(E, F)**. Forest plot showing the association between FLVCR1 expression and ESCC survival using univariate **(E)** and multivariate **(F)** analyses. (HR, hazard ratio; CI, confidence interval) **(G)** Representative images of FLVCR1 staining according to the T stage, N stage, and TNM stage of the ESCC patients (magnification, 200×). **(H)** Comparison of overall survival of ESCC patients with different FLVCR1 expression.

Based on TCGA data (http://ualcan.path.uab.edu), higher expression of FLVCR1 was detected in most carcinomas, including esophageal cancer (**Figure 1A**). In the present paper, a tissue microarray containing 31 paired ESCC and non-tumorous samples was stained with IHC to reveal the phenotype of FLVCR1 expression in ESCC. The results showed that the appearance of FLVCR1 was significantly higher in ESCC samples than in adjacent non-tumorous samples (**Figures 1B, C**). Moreover, the second set, including 77 matched ESCC and normal tissues, was stained with the IHC method to analyze the expression and prognosis of FLVCR1 in ESCC. As a result, the upregulated expression of FLVCR1 was noticed in 27 out of 77 ESCC cases (**Table 1**), but rarely detected in corresponding normal esophageal tissues. As shown in **Table 2** and **Figure 1D**, the results from 103 ESCC patients indicated that high FLVCR1 expression was significantly associated with lymph node-metastasis (N stage) and late tumor-node-metastasis (TNM stage), while there was no significant association between FLVCR1 expression and age, gender, differentiation grade, P53 positivity, Ki67 positivity, PDL-1 positivity or CD8 positivity. Univariate Cox regression analyses showed that the variables, including high FLVCR1 expression, and male sex, advanced T, N, and TNM stage, were associated with shorter overall survival (**Figure 1E**). However, multivariate Cox regression analysis revealed that FLVCR1 was not an independent prognostic factor for overall patient survival in patients with ESCC (**Figure 1F**). The representative staining results of ESCC patients with each T grade, N grade, and TNM stage were shown in **Figure 1G**. Furthermore, Kaplan-Meier analyses were used to analyze the correlation between FLVCR1 expression and overall survival in ESCC patients. ESCC patients with higher FLVCR1 expression exhibited a remarkably decreased overall survival (**Figure 1H**). Overall, these data indicated that the upregulation of FLVCR1 acts as a poor prognostic indicator of ESCC and might contribute to ESCC advancement.

**Table 1:** Differential expression of FLVCR1 in 77 paired of ESCC and adjacent tissues.

	FLVCR1 expression		*p*
	High	Low	
			0.000^***^
Tumor tissues	27	50	
Adjacent tissues	2	75	

***P < 0.001.

**Table 2:** The relationship between FLVCR1 expression status and clinicpathologic features of ESCC.

	Clinicpathologic features	Total	FLVCR1 expression		*p*
			Low	High	
Age (years)					0.116
	≤65	50	24	26	
	>65	52	33	19	
Gender					0.233
	Female	26	17	9	
	Male	77	40	37	
T stage					0.063
	T1/T2	19	14	5	
	T3/T4	78	39	39	
N stage					0.009^**^
	N0	46	32	14	
	N1/N2/N3	57	25	32	
TNM stage					0.008^**^
	I/II	47	32	15	
	III/IV	51	21	30	
Differentiation grade					0.411
	1/2	80	46	34	
	3/4	23	11	12	
P53					0.234
	Negative	24	11	13	
	Positive	53	32	21	
Ki67					0.723
	Negative	30	16	14	
	Positive	47	27	20	
PDL-1					0.896
	Negative	51	28	22	
	Positive	52	28	24	
CD8					0.896
	Negative	50	28	22	
	Positive	53	29	24	

T, tumor-metastasis; N, node-metastasis; TNM, tumor-node-metastasis; **P < 0.01.

### Knockdown of FLVCR1 Inhibits ESCC Cell Proliferation, and Colony Formation and Promotes Apoptosis

**Figure 2:**
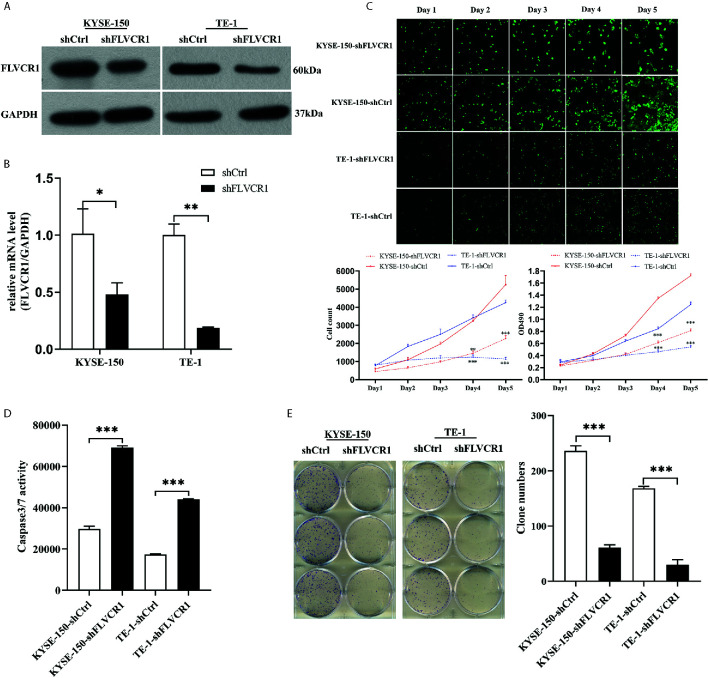
The effect of FLVCR1 on ESCC cell proliferation and apoptosis in vitro. **(A, B)** Protein and mRNA expression of FLVCR1 was significantly downregulated in shFLVCR1 groups compared with shCtrl groups. **(C)** The proliferation rate of TE‐1 and KYSE-150 cells was reduced by FLVCR1 knockdown, which was confirmed by the Celigo assay and MTT assay. **(D)** Knockdown of FLVCR1 remarkably enhanced caspase‐3/7 activity in the shFLVCR1 groups. **(E)** FLVCR1 knockdown decreased the colon numbers in cells of shFLVCR1 groups compared to cells of shCtrl groups, respectively. Data were presented as the mean±SD of three separate experiments. *P < 0.05, **P < 0.01, ***P < 0.001.

The RT‐PCR results showed that FLVCR1 mRNA levels were overexpressed in KYSE-150, TE‐1, Eca‐109, and EC9706 cells. In contrast, FLVCR1 expression was low in HEEC cells (**Supplementary 2**). TE‐1 and KYSE-150 cell lines were used to discover the function of FLVCR1 in the malignant characteristics of ESCC. ESCC cells with FLVCR1 knockdown (TE-1-shFLVCR1 and KYSE-150- shFLVCR1) were established, and the knockdown efficiency was confirmed by western blot and RT-PCR (**Figures 2A, B**). The Celigo and MTT assay data confirmed the inhibitory effect of FLVCR knockdown on the proliferation of ESCC cells (**Figure 2C**). As shown in **Figure 2D**, enhanced caspase‐3/7 activity was detected in ESCC cells with FLVCR1 knockdown, indicating that FLVCR1 knockdown could induce apoptosis. A colony formation assay was conducted to further verify that cell colony formation could be reduced by FLVCR1 knockdown in ESCC cells (**Figure 2E**).

### Knockdown of FLVCR1 Impedes ESCC Cell Migration and Invasion *In Vitro*

**Figure 3:**
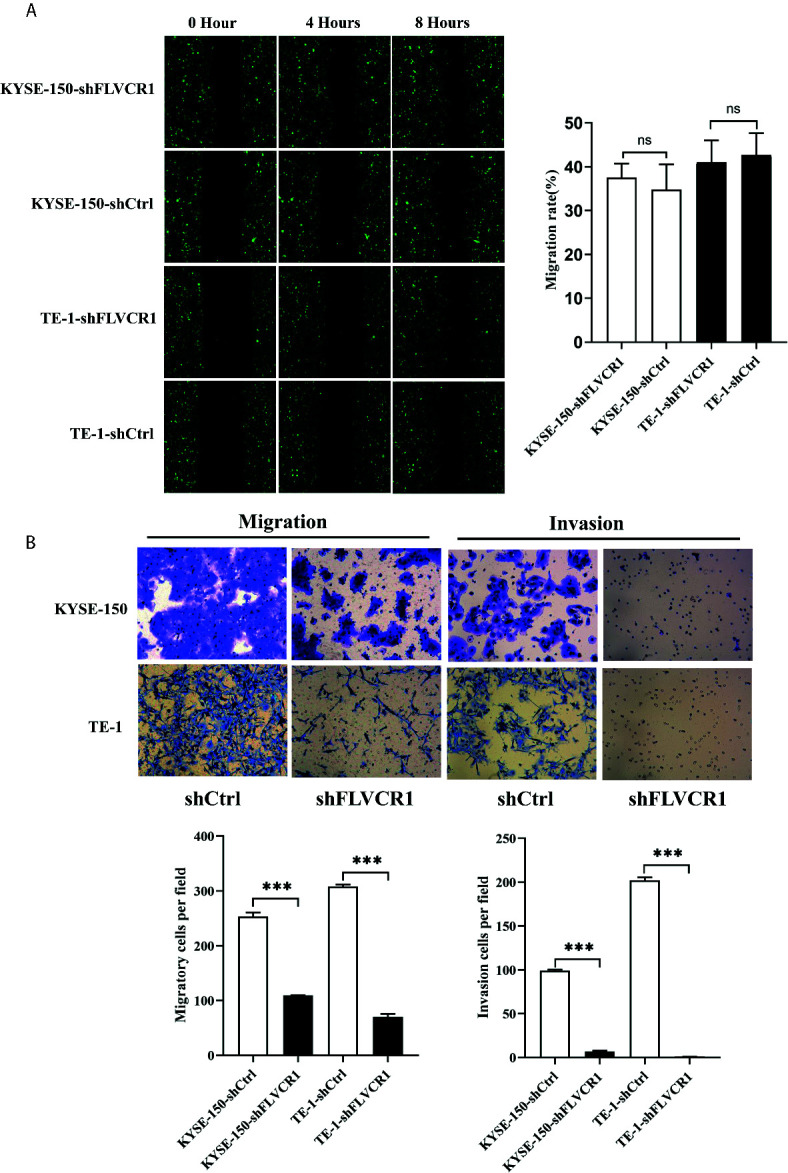
Knockdown of FLVCR1 decelerates ESCC cell migration and invasion. **(A)** Wound healing assay showed that inhibition of FLVCR1 did not significantly affect the motility of ESCC cells at 8 hours. **(B)** Transwell assays revealed that knockdown of FLVCR1 attenuated cell migration at 20 hours and reduced cell invasion at 48 hours. ***P < 0.001; ns, no significance.

Initially, we conducted a wound-healing assay to elucidate the role of FLVCR1 in the migration ability of ESCC cells. As shown in **Figure 3A**, inhibition of FLVCR1 did not have any effect on the migration of KYSE-150 and TE-1 cells compared to that of shCtrl groups after 8 h. Subsequently, we examined the role of FLVCR1 in the migratory ability of ESCC cells by using a Transwell migration assay. In contrast to the results of the wound healing assay, the negative effect of FLVCR1 knockdown on the migration ability of ESCC cells was confirmed in a Transwell migration assay (**Figure 3B**). Finally, a Transwell invasion assay was performed to assess the role of FLVCR1 in invasion. Similarly, the results of the Transwell invasion assay demonstrated that knockdown of FLVCR1 markedly attenuated the invasion of KYSE-150 and TE-1 cells compared with that of cells transfected with shCtrl (**Figure 3B**). In summary, these data revealed the negative effect of FLVCR1 knockdown on the migration and invasion of ESCC cells *in vitro*.

### Knockdown of FLVCR1 Suppresses ESCC Tumor Growth and Metastasis *In Vivo*

**Figure 4:**
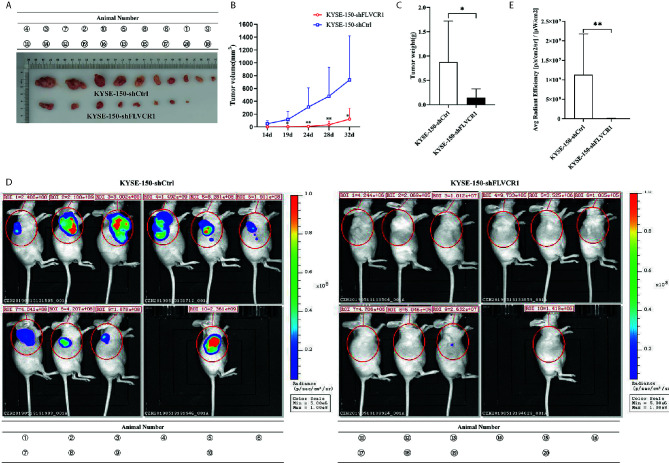
FLVCR1 knockdown retarded tumor growth in vivo. **(A)** KYSE-150-shFLVCR1 cells generated smaller tumors in nude mice than the KYSE-150-shCtrl cells. **(B)** The growth curve showed that FLVCR1 knockdown suppresses growth. **(C)** KYSE-150-shFLVCR1-formed tumors had significantly lighter weight than KYSE-150-shCtrl cell-formed tumors. **(D)** *In vivo* imaging of tumor-bearing mice. **(E)** Seriously weakened fluorescent radiant efficiency was found in KYSE-150-shFLVCR1 cell-injected mice compared to that of control mice. Data were represented as the mean ± SD. *P<0.05, **P<0.01.

**Figure 5:**
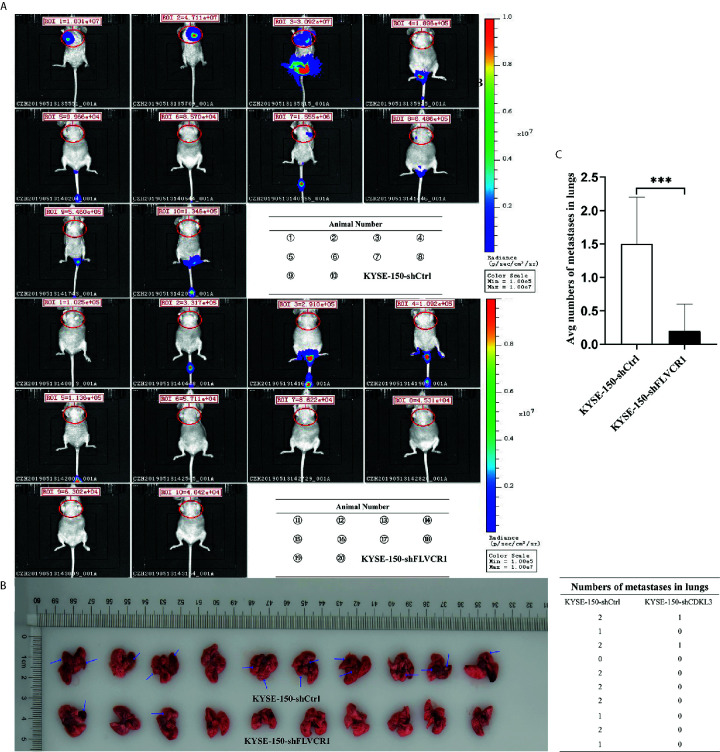
FLVCR1 knockdown reduced tumor metastasis *in vivo*. **(A)** *In vivo* imaging of tumor-bearing mice. **(B)** FLVCR1 knockdown suppressed the metastatic potential of KYSE-150 cells in nude mice. Red arrow: metastases **(C)** Severely weakened fluorescent radiant efficiency was found in KYSE-150-shFLVCR1 cells that formed metastases compared to that of control mice, ***P < 0.001.

The role of FLVCR1 in tumor growth and metastasis was further investigated in a tumor-bearing mouse model. As a result, inhibition of FLVCR1 slowed the tumor growth of KYSE-150 cells *in vivo* (**Figure 4A**). Furthermore, KYSE-150-shFLVCR1 cell-generated tumors had a smaller volume and lighter weight than KYSE-150-shCtrl cell-generated tumors (**Figures 4B, C**). The tumor-bearing mice were also photographed and analyzed by a non-invasive *in vivo* imaging system before sacrifice. The fluorescent radiant efficiency for the region of interests was calculated and demonstrated significant attenuation over control mice that were transplanted with KYSE-150-shCtrl cells (**Figures 4D, E**). We further investigated whether inhibition of FLVCR1 impeded metastasis *in vivo*. Seven weeks after tail vein injection, the transplanted mice were studied under an *in vivo* imaging system and carefully dissected. The lungs, livers, and tumors were removed for further analysis. Metastases were found in the lungs but not in the livers. Although the florescent intensity measurement is not significantly different between KYSE-150-shFLVCR1 and -shCtrl groups (as shown in **Supplementary Materials**), the frequency of metastasis to the lungs was decreased in mice injected with KYSE-150-shFLVCR1 cells as compared to KYSE-150-shCtrl cells (**Figures 5A–C**), suggesting a trend of functional impact of FLVCR1 on metastasis. Additionally, 5 out of 10(50%) mice implanted with shCtrl cells generated metastases in the groin, but none was found in the groin of the shFLVCR1 group. According to the above results, we confirmed the promoting function of FLVCR1 on tumor growth and metastasis *in vivo*.

### FLVCR1 Physically Interacted With CSE1L in ESCC Cells

**Figure 6:**
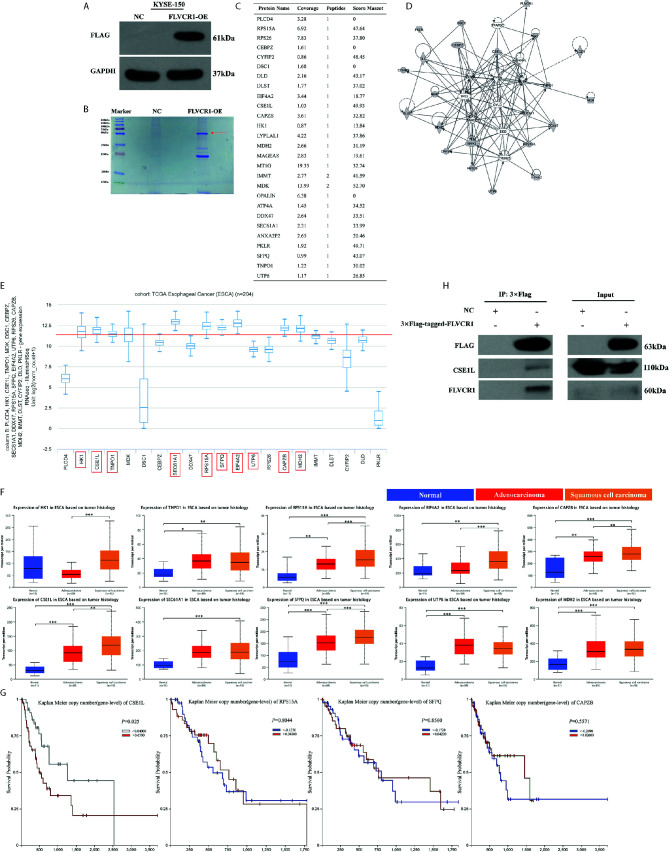
FLVCR1 interacts with CSE1L. **(A)** Western blotting was used to confirm that FLAG-FLVCR1 was successfully overexpressed in KYSE-150 cells. **(B)** Coomassie blue staining was used to detect immunoprecipitated Flag-FLVCR1-binding proteins. **(C)** MS analysis was used to identify target proteins interacting with FLVCR1, and a list of specific proteins that interact with FLVCR1 protein is shown. **(D)** The gene interaction network diagram demonstrated the interaction among multiple genes surrounding the regulation of FLVCR1. The 21 genes in gray represented the selected genes from MS analysis. Solid lines indicated direct interactions, while broken lines indicated indirect interactions. **(E)** The expression profile of 21 genes in the TCGA esophageal carcinoma cohort. Above the red line are the top-ten highly expressed genes. **(F)** The expression patterns of the top-ten highly expressed genes in the TCGA database according to tumor histology. **(G)** Comparison of overall survival of esophageal cancer patients with different CSE1L/RPS15A/SFPQ/CAPZB expression based on TCGA data. **(H)** The interaction between FLVCR1 and CSE1L identified by mass spectrometry analysis was confirmed by immunoprecipitation in KYSE-150 cells stably expressing FLVCR1. Red arrow: 3×FLAG-target. *P < 0.05, **P < 0.01, ***P < 0.001.

To explore the molecular mechanism by which FLVCR1 promotes proliferation and metastasis in ESCC cells, we sought to identify FLVCR1-interacting proteins in ESCC cells. We established stable KYSE-150 cell lines that constitutively overexpressed FLVCR1 containing a FLAG-tag (KYSE-150-FLVCR1-OE). The overexpression efficiency was confirmed by using a western blot assay with an anti-FLAG monoclonal antibody (**Figure 6A**). MS analysis identified 28 specific FLVCR1 interacting proteins based on the criteria of proteins that could be detected in the OE group (**Figures 6B, C**). Based on the results of MS and bioinformatics analysis, 21 genes (PLCD4, HK1, CSE1L, TNPO1, MDK, DSC1, CEBPZ, SEC61A1, DDX47, RPS15A, SFPQ, EIF4A2, UTP6, RPS26, CAPZB, MDH2, IMMT, DLST, CYFIP2, DLD, PKLR) were found. To investigate the interaction between FLVCR1 and these genes, bioinformatic analysis was applied to draw a diagram of the gene interaction network surrounding the regulation of FLVCR1 (**Figure 6D**). The top-ten highly expressed genes (HK1, CSE1L, TNPO1, SEC61A1, RPS15A, SFPQ, EIF4A2, UTP6, CAPZB, and MDH2) in esophageal cancer were further selected to analyze the differential expression in the esophageal cancer patient cohort in TCGA database using UCSC Xena (**Figure 6E**). Subsequently, UALCAN websites were employed to conduct expression profile analysis based on tumor histology (**Figure 6F**). The expression levels of CSE1L, RPS15A, SFPQ, and CAPZB in ESCC were significcantly higher than those in normal tissue and esophageal adenocarcinoma (EAC). Furthermore, we analyzed the survival prognosis of these genes in esophageal cancer patients *via* UCSC Xena. As shown in **Figure 6G**, lower expression of CSE1L was associated with longer overall survival in esophageal cancer patients. Therefore, CSE1L was selected as a potential downstream target gene of FLVCR1. The Co-IP assay illustrated that FLVCR1 and CSE1L could coprecipitate with each other in KYSE-150 cells (**Figure 6H**). Although anti-FLVCR1 antibody could not detect the target protein in the input sample, gray values of WB bands showed that the FLVCR1 level of the Flag-tagged transfected cell is higher than the control in the input sample after fusing the Flag tag (**Supplementary 3**).

### Restoration of CSE1L Expression Counteracts the Effects of FLVCR1 Knockdown

**Figure 7:**
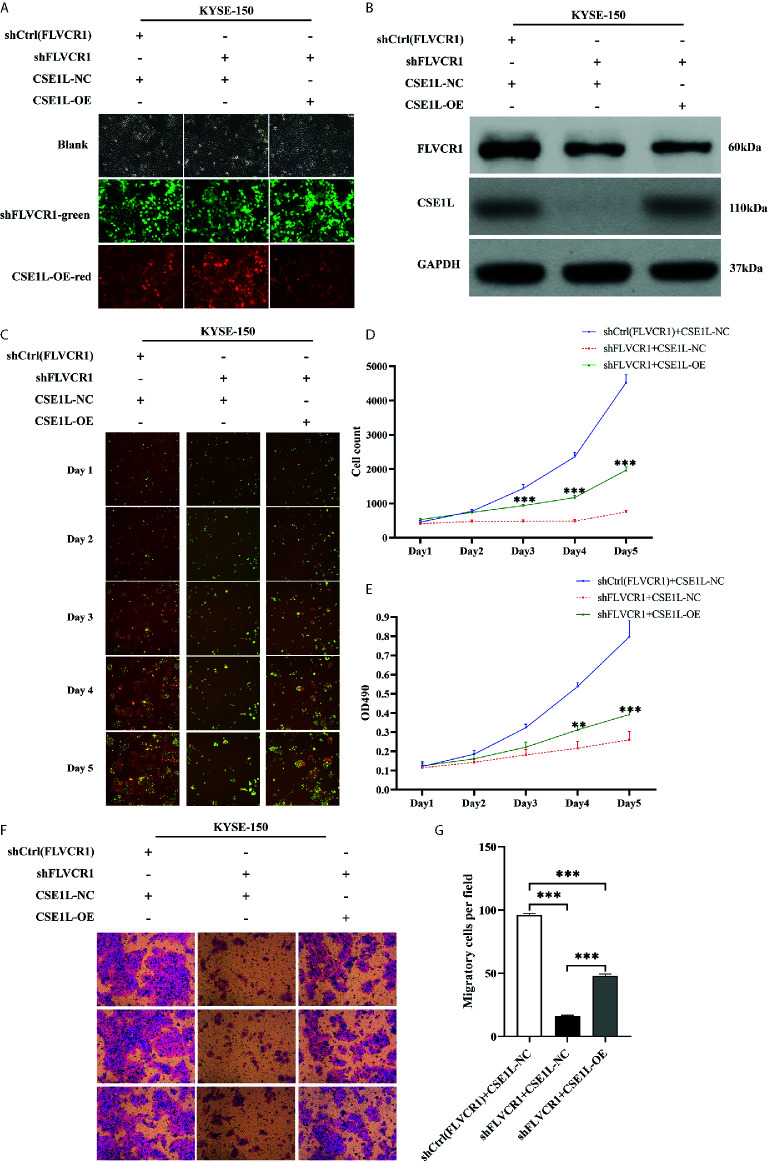
CSE1L re-expression partly counteracted the negative effect of FLVCR1 inhibition on the proliferation and migration of KYSE-150 cells. **(A)** KYSE-150 cells expressed green and red fluorescence after successful infection. Magnification 100×. **(B)**, Western blot analysis showed that the expression of CSE1L was restored in the shFLVCR1+CSE1L-OE group and downregulated in the ShFLVCR1+CSE1L-NC group. **(C–E)** Re-expressed CSE1L partially diminished the proliferation inhibitory effect of FLVCR1 knockdown compared to shFLVCR1+CSE1L-NC group. **(F, G)** Compared to the shFLVCR1+CSE1L-NC group, the migration ability was restored by ectopic expression of CSE1L. Data were represented as the mean ± SD. **P < 0.01, ***P < 0.001.

Next, rescue experiments were performed to verify the role of CSE1L in the impacts of FLVCR1 on the proliferation and migration of ESCC cells. The visualization of green and red fluorescence was detected to evaluate the infective efficiency under a fluorescence microscope, and more than 80% of KYSE-150 cells were successfully transfected with the recombinant plasmids (**Figure 7A**). The results of western blot analysis suggested that FLVCR1 knockdown substantially downregulated the expression of CSE1L in KYSE-150 cells (**Figure 7B**). However, CSE1L was highly re-expressed in KYSE-150 cells cotransfected with FLVCR1 knockdown and ectopic-expression of CSE1L (**Figure 7B**). For re-expressed CSE1L, the growth inhibitory effect of FLVCR1 knockdown on KYSE-150 cells was attenuated (**Figures 7C–E**), and the reduced migratory capability of FLVCR1 knockdown on ESCC cells was also abolished (**Figures 7F, G**).

## Discussion

Recent studies have focused on the crucial role of FLVCR1 in tumor promotion. For instance, upregulated FLVCR1 promoted the cellular proliferation of neuroblastoma and synovial sarcoma (9, 10). Aberrant expression of FLVCR1 was correlated with aggressive tumor stage and poor survival in hepatocellular carcinoma (11). The current study demonstrated a pivotal role and potential mechanism of FLVCR1 in regulating the proliferation and migration of ESCC for the first time. Our results exhibited that downregulation of FLVCR1 remarkably induced apoptosis and suppressed the proliferation, migration, invasion, and colony formation of ESCC cells. These findings indicated the function of FLVCR1 in the formation and progression of the malignant phenotype of ESCC.

Interestingly, wound healing assays showed that FLVCR1 knockdown had not begun to affect the migration of ESCC cells after as little as 8 hours. The inhibition of FLVCR1 knockdown on ESCC migration was investigated by a Transwell migration assay after more than 20 hours of incubation, suggesting that FLVCR1 may have a time-dependent effect on the cellular migration of ESCC. Tumor metastasis is a comprehensive process during which cells detach from the primary site, disseminate throughout the body, and form new metastases by adhesion. By wound healing assay, cells moved to the wound by laterally creeping and displayed a horizontal migration. However, a Transwell migration assay showed stereoscopic migration (12, 13). Also, wound healing assays showed the motility of clusters of cells, while Transwell migration assays revealed the migratory ability of a single cell (14, 15). Furthermore, the vital role of FLVCR1 in tumorigenesis and migration of ESCC was confirmed in a tumor-bearing mouse model.

Next, Co-IP and LC-MS were utilized to explore the potential FLVCR-binding protein, which might influence the proto-oncogene FLVCR1. In this study, CSE1L was identified as a novel downstream target of FLVCR1 and implicated in the regulation of tumor growth and migration in ESCC. CSE1L, a human homolog of the yeast chromosome segregation protein, is up-regulated in multiple types of carcinomas, including colorectal cancer, lung cancer, breast cancer, and so on (16). CSE1L was uncovered to promote the tumor progression by affecting cell proliferation, apoptosis, and invasion of colorectal cancer (17). CSE1L silencing could induce apoptosis and repress the proliferation and invasion of gastric cancer cells by regulating the PI3K/Akt/mTOR and MEK/ERK signaling pathways (18). In addition, a recent study revealed that the abnormal expression of CSE1L was correlated with neoplastic progression in Barrett’s esophagus (19). However, the role of CSE1L has not been studied in esophageal cancer.

CSE1L was one of the proteins that interacted with FLVCR1 based on our data. Therefore it was selected as an essential candidate for further ESCC research. The results of Co-IP showed the interaction between CSE1L and FLVCR1, indicating that CSE1L might be required for FLVCR1-mediated tumor promotion in ESCC. CSE1L expression was downregulated by FLVCR1 knockdown in KYSE-150 cells. The inhibition of ESCC induced by FLVCR1 knockdown can be partly restored by CSE1L overexpression. However, more comprehensive and in-depth studies are needed in our next plan to explore the precise molecular mechanisms involved in interactions between FLVCR1 and CSE1L. Besides, CSE1L can be detected not only in tumor tissues but also in body fluids, especially in the blood, indicating that CSE1L can be used as a tumor serum biomarker (20–22). Therefore, our further research plan is to investigate the diagnostic and prognostic value of serum CSE1L in ESCC.

Moreover, there is no information about the indicative function of FLVCR1 in ESCC prognosis in previous reports. In this paper, the correlation between FLVCR1 expression and the clinicopathological features of ESCC patients was identified by immunohistochemical analysis. The levels of FLVCR1 expression in ESCC tissues were significantly higher than those in corresponding normal tissues. Additionally, the upregulation of FLVCR1 expression was positively correlated with the aggressive characteristics of ESCC, including advanced lymph node metastasis and advanced TNM stage. In a previous study, overexpression of FLVCR1 was known to be strongly associated with poorer survival outcomes of patients with hepatocellular carcinoma (11). In this paper, FLVCR1 was not an independent prognostic factor in ESCC, but a prognostic marker for worse survival in ESCC patients.

Furthermore, another analysis was conducted to evaluate the relationship between FLVCR1 expression status and the expression of p53, Ki67, PDL-1a, and CD8. Ki67, a proliferation indicator, is related to the poor prognosis of patients with various tumors (23–25). P53 is the most frequently mutated gene in ESCC, and p53 mutation shows excellent prediction performance on treatment response and overall survival in ESCC (26–28). Abnormal PDL-1 expression has been identified in various human malignancies, and antibody-mediated blockade of PDL-1 shows efficient antitumor activity (29). The PD−1/PDL-1 pathway can prevent effective antitumor immunity by downregulating the function of CD8+ T lymphocytes, so ESCC patients who are PDL-1−positive have a poorer prognosis than negative patients (30). However, our results showed that there was no association between FLVCR1 expression and the positive expression of Ki67, p53, PDL-1, and CD8. Our data on the expression of Ki67, p53, PDL-1, and CD8 from IHC are qualitative results, and more accurate quantitative analysis should be performed in further studies. In addition, CD8^+^ T cells can be observed in peripheral blood (peripheral CD8^+^ T cells) and tumor microenvironments (CD8^+^ tumor-infiltrating lymphocytes). CD8^+^ tumor-infiltrating lymphocytes (CD8^+^ TILs) were found to be positively associated with the prognosis of ESCC patients (31, 32). FLVCR1, a major facilitator superfamily metabolite transporter, has been reported to contribute to the development and survival of peripheral CD4^+^ and CD8^+^ T cells (33). Studies on the relationship between FLVCR1 and CD8^+^ TILs are still lacking. Therefore, we will focus on the relationship between FLVCR1 and tumor-infiltrating CD8^+^ T cells or peripheral CD8^+^ T cells in ESCC in further studies.

In summary, these results indicate that upregulated FLVCR1 has been verified as a poor prognostic indicator in patients with ESCC. Functional experiments confirmed that FLVCR1 may function as an oncogene in ESCC by promoting cell growth, migration, and invasion and repressing apoptosis. We preliminarily confirmed the involvement of CSE1L in the effects of FLVCR1 on the cellular proliferation and migration of ESCC, indicating that FLVCR1-targeting treatment may be promising in the therapies of patients with ESCC.

## Author's Note

Parts of this work have been presented as an abstract at European-Society-for-Medical-Oncology (ESMO) 21st World Congress on Gastrointestinal Cancer and the ESMO Virtual Congress 2020.

## Data Availability Statement

The original contributions presented in the study are included in the article/**Supplementary Material**. Further inquiries can be directed to the corresponding author.

## Ethics Statement

The studies involving human participants were reviewed and approved by The Institutional Review Board of Taizhou Hospital. The patients/participants provided their written informed consent to participate in this study. The animal study was reviewed and approved by Institutional Animal Care and Use Committee (IACUC) of Wenzhou Medical University.

## Author Contributions

SZ, WY, and HY conceived and participated in the design of the study. The manuscript was written and revised by SZ and MZ. CZ recruited samples. SZ, CZ, and YM performed all the experimental work. SZ, WY, and HY participated in data analysis. All authors contributed to the article and approved the submitted version.

## Funding

This study was supported by National Natural Science Foundation of China (NSFC 81872458), Natural Science Foundation of Zhejiang Province (LY19H160017), and Medical Science and Technology Project of Zhejiang Province (2020KY358).

## Conflict of Interest

The authors declare that the research was conducted in the absence of any commercial or financial relationships that could be construed as a potential conflict of interest.

## References

[1] Siegel RL Miller KD Jemal A. Cancer statistics, 2018. CA Cancer J Clin (2018) 68:7–30. 10.3322/caac.21442 29313949

[2] Lin Y Totsuka Y He Y Kikuchi S Qiao Y Ueda J. Epidemiology of Esophageal Cancer in Japan and China. J Epidemiol (2013) 23:233–42. 10.2188/jea.je20120162 PMC3709543 23629646

[3] Chen W Zheng R Baade PD Zhang S Zeng H Bray F. Cancer statistics in China, 2015. CA Cancer J Clin (2016) 66:115–32. 10.3322/caac.21338 26808342

[4] Zeng H Zheng R Guo Y Zhang S Zou X Wang N. Cancer survival in China, 2003-2005: A population-based study. Int J Cancer (2015) 136:1921–30. 10.1002/ijc.29227 25242378

[5] Wei L Yan N Sun L Bao C Li D. Interplay between the NF−κB and hedgehog signaling pathways predicts prognosis in esophageal squamous cell carcinoma following neoadjuvant chemoradiotherapy. Int J Mol Med (2018) 41:2961–67. 10.3892/ijmm.2018.3447 29393402

[6] Chiabrando D Vinchi F Fiorito V Mercurio S Tolosano E. Heme in pathophysiology: a matter of scavenging, metabolism and trafficking across cell membranes. Front Pharmacol (2014) 5:61. 10.3389/fphar.2014.00061 24782769 PMC3986552

[7] Wiel C Le Gal K Ibrahim MX Jahangir CA Kashif M Yao H. BACH1 Stabilization by Antioxidants Stimulates Lung Cancer Metastasis. Cell (2019) 178:330–45. 10.1016/j.cell.2019.06.005 31257027

[8] Sohoni S Ghosh P Wang T Kalainayakan SP Vidal C Dey S. Elevated Heme Synthesis and Uptake Underpin Intensified Oxidative Metabolism and Tumorigenic Functions in Non-Small Cell Lung Cancer Cells. Cancer Res (2019) 79:2511–25. 10.1158/0008-5472 30902795

[9] Chiabrando D Castori M di Rocco M Ungelenk M Gießelmann S Di Capua M. Mutations in the Heme Exporter FLVCR1 Cause Sensory Neurodegeneration with Loss of Pain Perception. PloS Genet (2016) 12:e1006461. 10.1371/journal.pgen.1006461 27923065 PMC5140052

[10] Peng C Song Y Chen W Wang X Liu X Wang F. FLVCR1 promotes the proliferation and tumorigenicity of synovial sarcoma through inhibiting apoptosis and autophagy. Int J Oncol (2018) 52:1559–68. 10.3892/ijo.2018.4312 29532854

[11] Shen Y Li X Zhao B Xue Y Wang S Chen X. Iron metabolism gene expression and prognostic features of hepatocellular carcinoma. J Cell Biochem (2018) 119:9178–204. 10.1002/jcb.27184 30076742

[12] Mccord RP Golloshi R. Abstract 5373: 3D genome architecture changes during cancer cell migration and metastasis. Cancer Res (2017) 77(13 Supplement):5373–73. 10.1158/1538-7445.AM2017-5373

[13] Yang Y Hu S Xu X Li J Liu A Han J. The Vascular Endothelial Growth Factors-Expressing Character of Mesenchymal Stem Cells Plays a Positive Role in Treatment of Acute Lung Injury In Vivo. Mediators Inflamm (2016) 2016:2347938. 10.1155/2016/2347938 27313398 PMC4895047

[14] Rodriguez LG Wu X Guan JL. Wound-healing assay. Methods Mol Biol (2005) 294:23–9. 10.1385/1-59259-860-9:023 15576902

[15] Justus CR Leffler N Ruiz-Echevarria M Yang LV. In vitro cell migration and invasion assays. J Vis Exp (2014) 1:51046. 10.3791/51046 PMC4186330 24962652

[16] Jiang MC. CAS (CSE1L) signaling pathway in tumor progression and its potential as a biomarker and target for targeted therapy. Tumour Biol (2016) 37:13077–90. 10.1007/s13277-016-5301-x 27596143

[17] Alnabulsi A Agouni A Mitra S Garcia-Murillas I Carpenter B Bird S. Cellular apoptosis susceptibility (chromosome segregation 1-like, CSE1L) gene is a key regulator of apoptosis, migration and invasion in colorectal cancer. J Pathol (2012) 228(4):471–81. 10.1002/path.4031 22450763

[18] Li Y Yuan S Liu J Wang Y Zhang Y Chen X. CSE1L silence inhibits the growth and metastasis in gastric cancer by repressing GPNMB via positively regulating transcription factor MITF. J Cell Physiol (2020) 235:2071–79. 10.1002/jcp.29107 31347172

[19] Jiang K Neill K Cowden D Klapman J Eschrich S Pimiento J. Expression of CAS/CSE1L, the Cellular Apoptosis Susceptibility Protein, Correlates With Neoplastic Progression in Barrett’s Esophagus. Appl Immunohistochem Mol Morphol (2018) 26:552–6. 10.1097/PAI.0000000000000464 PMC5466515 27941559

[20] Tai CJ Liao CF Su TC Shen KH Chang CC Lin SH. Presence of CSE1L protein in urine of patients with urinary bladder urothelial carcinomas. Int J Biol Markers (2012) 27:e280–4. 10.5301/JBM.2012.9310 22653741

[21] Liao CF Lin SH Chen HC Tai CJ Chang CC Li LT. CSE1L, a novel microvesicle membrane protein, mediates Ras-triggered microvesicle generation and metastasis of tumor cells. Mol Med (2012) 18:1269–80. 10.2119/molmed.2012.00205 PMC3521793 22952058

[22] Lee WR Shen SC Shih YH Chou CL Tseng JT Chin SY. Early decline in serum phospho-CSE1L levels in vemurafenib/sunitinib-treated melanoma and sorafenib/lapatinib-treated colorectal tumor xenografts. J Transl Med (2015) 13:191. 10.1186/s12967-015-0553-6 26070816 PMC4467675

[23] Peng Y Wang L Gu J. Elevated preoperative carcinoembryonic antigen (CEA) and Ki67 is predictor of decreased survival in IIA stage colon cancer. World J Surg (2013) 37:208–13. 10.1007/s00268-012-1814-7 23052808

[24] Li H Han X Liu Y Liu G Dong G. Ki67 as a predictor of poor prognosis in patients with triple-negative breast cancer. Oncol Lett (2015) 9:149–52. 10.3892/ol.2014.2618 PMC4246616 25435949

[25] Turkel Kucukmetin N Cicek B Saruc M Ersoy O Vardareli E Onder O. Ki67 as a prognostic factor for long-term outcome following surgery in gastrointestinal stromal tumors. Eur J Gastroenterol Hepatol (2015) 27:1276–80. 10.1097/MEG.0000000000000454 26275084

[26] Liu X Zhang M Ying S Zhang C Lin R Zheng J. Genetic alterations in esophageal tissues from squamous dysplasia to carcinoma. Gastroenterology (2017) 153:166–77. 10.1053/j.gastro.2017.03.033 28365443

[27] Kandioler D Schoppmann SF Zwrtek R Kappel S Wolf B Mittlböck M. The biomarker TP53 divides patients with neoadjuvantly treated esophageal cancer into 2 subgroups with markedly different outcomes. A p53 Research Group study. J Thorac Cardiovasc Surg (2014) 148:2280–6. 10.1016/j.jtcvs.2014.06.079 25135238

[28] Zhao Z Wang P Gao Y He J. The high expression instead of mutation of p53 is predictive of overall survival in patients with esophageal squamous-cell carcinoma: a meta-analysis. Cancer Med (2017) 6:54–66. 10.1002/cam4.945 27882726 PMC5269704

[29] Brahmer JR Tykodi SS Chow LQ Hwu WJ Topalian SL Hwu P. Safety and activity of anti-PD-L1 antibody in patients with advanced cancer. N Engl J Med (2012) 366:2455–65. 10.1056/NEJMoa1200694 PMC3563263 22658128

[30] Leng C Li Y Qin J Ma J Liu X Cui Y. Relationship between expression of PD-L1 and PD-L2 on esophageal squamous cell carcinoma and the antitumor effects of CD8^+^ T cells. Oncol Rep (2016) 35:699–708. 10.3892/or.2015.4435 26718132

[31] Han L Gao QL Zhou XM Shi C Chen GY Song YP. Characterization of CD103(+) CD8(+) tissue-resident T cells in esophageal squamous cell carcinoma: may be tumor reactive and resurrected by anti-PD-1 blockade. Cancer Immunol Immunother (2020) 69:1493–504. 10.1007/s00262-020-02562-3 PMC11027643 32285170

[32] Nishimura J Tanaka H Yamakoshi Y Hiramatsu S Tamura T Toyokawa T. Impact of tumor-infiltrating LAMP-3 dendritic cells on the prognosis of esophageal squamous cell carcinoma. Esophagus (2019) 16:333–44. 10.1007/s10388-019-00669-w 30968254

[33] Philip M Funkhouser SA Chiu EY Phelps SR Delrow JJ Cox J. Heme exporter FLVCR is required for T cell development and peripheral survival. J Immunol (2015) 194:1677–85. 10.4049/jimmunol.1402172 PMC4323866 25582857

