# Supplementary material for: FLVCR1 Predicts Poor Prognosis and Promotes Malignant Phenotype in Esophageal Squamous Cell Carcinoma via Upregulating CSE1L
Source: Front Oncol. 2021 Mar 25;11:660955. doi: 10.3389/fonc.2021.660955 (PMC8027484; doi:10.3389/fonc.2021.660955)

Supplementary_1 The representative photographs of the FLVCR1 IHC staining with a score according to the percentage and intensity of positively stained cells.


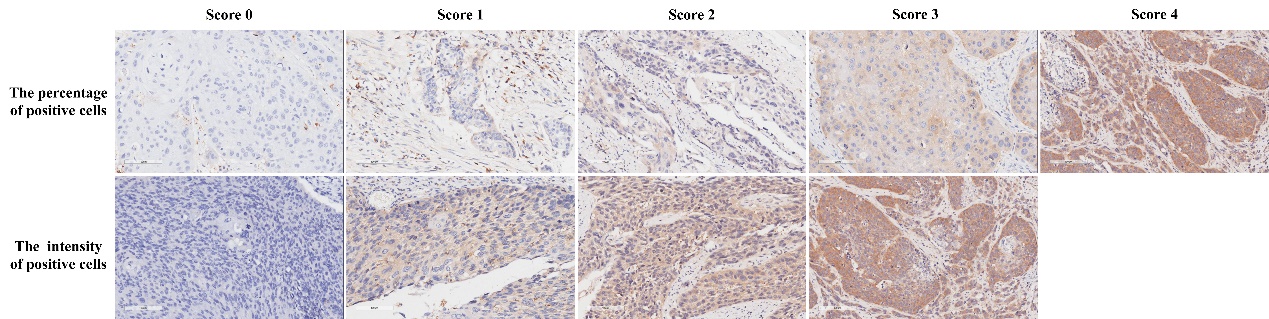

Supplement: Supplementary file 1 [file Table_1.docx]
