# Supplementary material for: FLVCR1 Predicts Poor Prognosis and Promotes Malignant Phenotype in Esophageal Squamous Cell Carcinoma via Upregulating CSE1L
Source: Front Oncol. 2021 Mar 25;11:660955. doi: 10.3389/fonc.2021.660955 (PMC8027484; doi:10.3389/fonc.2021.660955)
Supplement: Supplementary file 2 [file Table_2.docx]

**Part I**

**Preparation of target gene RNA interference lentiviral vector**

**Experimental objectives**

To design RNA interference target sequence and construct target gene RNA interference lentiviral vector using FLVCR1 gene as template.

**Experimental procedure**

After completing the design of RNA interference target, single-stranded DNA oligo containing the interference sequence is synthesized and annealed to produce double-stranded DNA; the ligated product is then directly ligated into the enzymatically cleaved lentiviral vector through the enzymatic cleavage sites at both ends; the ligated product is transferred into the prepared E. coli receptor cells, the positive recombinant is identified by PCR, sent to sequencing for verification, and the clone with the correct sequencing result is subjected to plasmid extraction.

**Experimental materials**

**1. Experimental plasmids**

1. Experimental plasmids

Vector number: GV115

Component sequence: hU6-MCS-CMV-EGFP

Control insert sequence: TTCTCCGAACGTGTCACGT

Tool vector mapping：

**
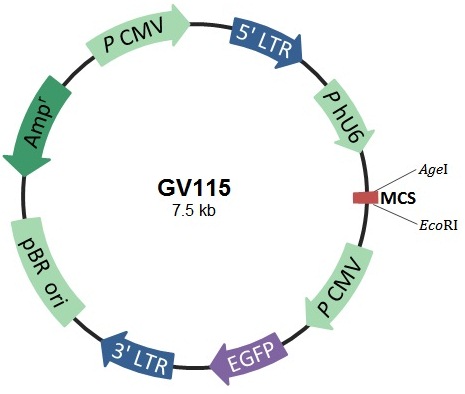
**

**2. Experimental strains**

TOP10 E. coli receptor cells (TIANGEN, Cat. #CB104-03)

**3. Experimental reagents**

**3.1 Enzyme reagents**

| **Reagents** | **Supplier** | **Cat. #** |
| --- | --- | --- |
| Age I | NEB | R3552L |
| EcoRI | NEB | R3101L |
| CutSmart Buffer | NEB | B7204S |
| Taq Plus DNA Polymerase | Vazyme | P201-D3 |
| T4 DNA Ligase | Fermentas | EL0016 |

**3.2 Other Reagents**

| **Reagent Name** | **Supplier** | **Cat. #** |
| --- | --- | --- |
| dsDNA oligo | Shanghai Generay Biotech Co.,Ltd |  |
| PCR primer（R&F） | Shanghai Generay Biotech Co.,Ltd |  |
| TIANgel Midi Purification Kit | TIANGEN | DP209-03 |
| EndoFree Maxi Plasmid Kit | TIANGEN | DP117 |
| Tryptone | OXOID | LP0042 |
| Yeast Extract | OXOID | LP0021 |
| NaCl | Sangon Biotech | 0241-500g |
| Tris | Sangon Biotech | T0826-500g |
| EDTA | Sangon Biotech | E0105-500g |
| Ampicillin，sodium salt | Genebase | A100339-0025 |
| Agarose | SBS Genetech | GA4-100 |
| 250bp-II DNA Ladder | shanghai Generay Biotech Co.,Ltd | DL2502 |
| GeneRuler 1kb DNA Ladder | Thermo Scientific | SM0311 |
| DNA sequencing | Major BioTech | ABI3733 |

**3.3 Experimental apparatus**

| Instrument name | **Supplier** | **Cat. #** |
| --- | --- | --- |
| Digital display type voltage stabilized electrophoresis instrument | Tanon | EPS200 |
| Gel imager | Tanon | Tanon-2500 |
| Bacterial shaker | Hualida Experimental Equipment Co. | HI-9211K |
| Water-tight incubator | Shanghai Yiheng Scientific Instruments Co. | GHP-9080 |
| PCR instrument | Applied Biosystems | 2720 thermal cycler |
| High-speed centrifuge | Thermo Scientific | Legend Micro 17 |
| Nanodrop 2000 | Thermo Scientific |  |
| Gilson Pipette | Gilson Corporation |  |

**4. Preparation of experimental reagents**

**4.1 Annealing buffer (pH=7.5-8.0)**

| **Drug** | **Concentration** |
| --- | --- |
| Tris | 10mM |
| NaCl | 50mM |
| EDTA | 1mM |

**4.2 LB liquid medium (100 ml, pH=7.0)**

| **Drug** | **Weight** | **Percentage** |
| --- | --- | --- |
| Yeast Extract | 0.5g | 0.5% |
| Tryptone | 1g | 1% |
| NaCl | 1g | 1% |

The content of ampicillin in LB medium containing ampicillin is 100μg/ml.

**4.3 LB solid medium (100 ml, pH=7.0)**

| **Drug** | **Weight** | **Percentage** |
| --- | --- | --- |
| Yeast Extract | 0.5g | 0.5% |
| Tryptone | 1g | 1% |
| NaCl | 1g | 1% |
| Agarose | 1.5g | 1.5% |

The content of ampicillin in LB medium containing ampicillin is 100μg/ml.

**RNA interference target design and double-stranded DNA oligo preparation**

1. Gene Information

| **Symbol** | **Accession#** | **Species** | **Full name** |
| --- | --- | --- | --- |
| FLVCR1 | NM_014053 | Homo sapiens | Homo sapiens feline leukemia virus subgroup C cellular receptor 1 (FLVCR1), mRNA |

2. RNA interference target design

According to the RNA interference sequence design principles, multiple 19-21nt RNA interference target sequences were designed using the FLVCR1 gene as a template. After evaluation and determination by the design software, the following sequences were selected as interference targets.

| **Target number** | **Internal number** | **Target sequence** | **GC%** |
| --- | --- | --- | --- |
| pSC-1 | psc14021 | CATCAGACTATGGTCCTAA | 42.10% |

1. DNA oligo sequence synthesis

The shRNA interference sequence was designed based on the selected target sequence, and suitable restriction endonuclease sites were added at both ends to complete the vector construction. In addition, a TTTTT termination signal is added at the 3' end of the forward strand, and a complementary sequence is added at the 5' end of the reverse strand. After the design was completed, single-stranded DNA oligo was synthesized by Generay Biotech Co.,Ltd.

| **Internal numbering** | **5' additional base** |  | **STEM** | **Loop** | **STEM** | **3’ additional base** |
| --- | --- | --- | --- | --- | --- | --- |
| psc14021-1 | CCGG |  | caCATCAGACTATGGTCCTAA | CTCGAG | TTAGGACCATAGTCTGATGTG | TTTTTG |
| psc14021-2 | AATTCAAAAA |  | caCATCAGACTATGGTCCTAA | CTCGAG | TTAGGACCATAGTCTGATGTG |  |

＊CCGG：AgeI digest site; AATTC: EcoRI digest site; G: EcoRI digest site complementary sequence.

4. Double-stranded DNA oligo preparation

The synthesized single-stranded DNA oligo dry powder was dissolved in annealing buffer (final concentration 20μM) and water-bath at 90°C for 15min. After natural cooling to room temperature, the double strand with sticky ends was formed.

**Preparation of linearized vector**

Prepare 50μl reaction system according to NEB instructions and linearize GV115 vector by double digestion with AgeI and EcoRI.

| **Reagents** | **Amount used** |
| --- | --- |
| Vector (1 µg/µl) | 2μl |
| CutSmart Buffer | 5μl |
| AgeI (10 U/µl) | 1μl |
| EcoRI (10 U/µl) | 1μl |
| H_2_O | Up to 50μl |

The reaction was carried out at 37°C (optimum temperature) for 1 h, after which the target fragments were recovered by cutting the gel.

**Electrophoresis sample loading instructions**

Lane 1: 1kb Marker: 10kb, 8kb, 6kb, 5kb, 4kb, 3.5kb, 3kb, 2.5 kb, 2kb, 1.5kb, 1kb, 750bp, 500bp, 250bp from top to bottom

Lane 2: Vector plasmid after linearization by double digestion of Age I and EcoR I

Lane 3: Vector plasmid without enzyme digestion

**Agarose gel electrophoresis images**

**1 2 3**


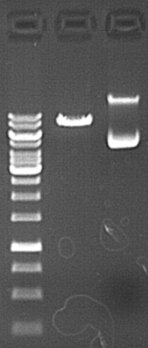


Note: Due to the company's quality control needs, after a large number of vector digestions, the quality-checked enzyme sections will be saved for multiple users' vector constructs, so this image will appear in multiple users' reports. If you need to use this image elsewhere, please mark in the experimental material: GV115 vector, AgeI/EcoRI digestion, purchased from Shanghai Genechem Co.

RNA interference lentiviral vector construction

1. ligation

Prepare 20 μl reaction system according to Fermentas T4 DNA Ligase instructions and ligate the double-stranded DNA oligo to the linearized vector.

| **Reagents** | **usage** |
| --- | --- |
| Linearized Vector (100 ng/µl) | 1μl |
| Insert (100 ng/µl) | 1μl |
| 10×T4 DNA ligase Buffer | 2μl |
| T4 DNA ligase | 1μl |
| H_2_O | Up to 20μl |

The reaction was carried out at 16℃ for 1h-3h, and the ligated product was named psc14021, followed by transformation experiments.

1. Transformation

Transform the ligation product into E. coli receptor cells with the following detailed steps：

1. Add 10μl of ligation product psc14021 to 100μl of E. coli receptor cells and ice bath for 30min.
2. Heat stimulate at 42°C for 90sec and ice bath for 2min.
3. Add 500 µL of LB liquid medium without antibiotics and incubate for 1hr at 200rpm in a shaker at 37°C.
4. Take 150 µl of bacterial solution and spread it evenly on LB solid medium containing Amp and incubate overnight at 37°C in an incubator.

3. PCR identification of positive clones

3.1 Schematic diagram of RNA interference vector construction and positive clone identification


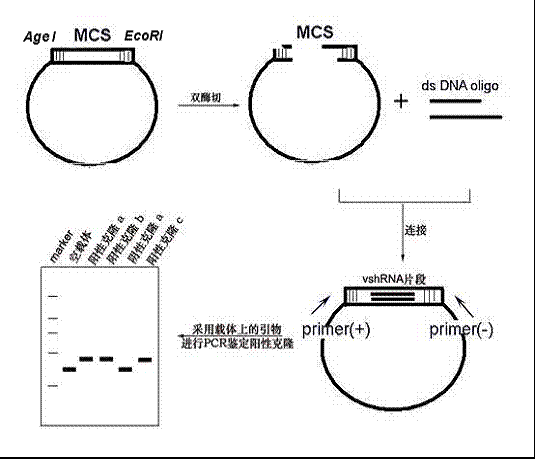


3.2 Primers

| **Primer name** | **Primer sequence (5' → 3')** |
| --- | --- |
| Identification primer-F | CCTATTTCCCATGATTCCTTCATA |
| Identification primer-R | GTAATACGGTTATCCACGCG |

3.2 PCR amplification

Prepare 20μl PCR reaction system according to the table below, pick a single colony as template with a sterile gun and carry out PCR amplification, the reaction conditions are: 94℃ for 3min; 94℃ for 30s, 55℃ for 30s, 72℃ for 30s, 22 cycles; 72℃ for 5min. After PCR, take 5μl of product and detect the bands by 1% agarose gel electrophoresis.

| **Reagents** | **Usage** |
| --- | --- |
| Taq Plus DNA Polymerase | 0.2μl |
| 10 x Buffer | 2μl |
| Identification primer-F | 0.4μl |
| Identification primer-R | 0.4μl |
| Template | - |
| H_2_O | Up to 20μl |

**Electrophoresis sample loading instructions**

Lane 1: Negative control (ddH2O) to exclude false positive results due to exogenous nucleic acid contamination in the system

Lane 2: self-linked control (empty vector self-linked control)

Lane 3: 250bp Marker: 5kb, 3kb, 2kb, 1.5kb, 1kb, 750bp, 500bp, 250bp, 100bp from top to bottom

Lanes 4-8: monoclonal psc14021-1,2,3,4,5

**Agarose gel electrophoresis images**

**1 2 3 4 5 6 7 8**


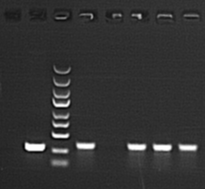


PCR band size

PCR fragment size of positive clone with shRNA fragment ligated: 380bp;

The PCR fragment size of the empty vector clone without shRNA fragment ligated into it: 307bp.

Thus, psc14021-1,3,4,5 were identified as positive clones, and the clones with correct identification results were saved and sequenced.

4. Analysis of positive clone sequencing results

The positive clones were sequenced with the identification primer-F, and the clones with identical sequencing results to the target sequences were selected for the next experiment.

Sequencing results of psc14021

TGACTGTAAACACAAAGATATTAGTACAAAATACGTGACGTAGAAAGTAATAATTTCTTGGGTAGTTTGCAGTTTTAAAATTATGTTTTAAAATGGACTATCATATGCTTACCGTAACTTGAAAGTATTTCGATTTCTTGGCTTTATATATCTTGTGGAAAGGACGAAACACCGGCACATCAGACTATGGTCCTAACTCGAGTTAGGACCATAGTCTGATGTGTTTTTGAATTCTCGACCTCGAGACAAATGGCAGTATTCATCCACGAATTCGGATCCATTAGGCGGCCGCGTGGATAACCGTATTACCGCCATGCATTAGTTATTAATAGTAATCAATTACGGGGTCATTAGTTCATAGCCCATATATGGAGTTCCGCGTTACATAACTTACGGTAAATGGCCCGCCTGGCTGACCGCCCAACGACCCCCGCCCATTGACGTCAATAATGACGTATGTTCCCATAGTAACGCCAATAGGGACTTTCCATTGACGTCAATGGGTGGAGTATTTACGGTAAACTGCCCACTTGGCAGTACATCAAGTGTATCATATGCCAAGTACGCCCCCTATTGACGTCAATGACGGTAAATGGCCCGCCTGGCATTATGCCCAGTACATGACCTTATGGGACTTTCCTACTTGGCAGTACATCTACGTATTAGTCATCGCTATTACCATGGTGATGCGGTTTTGGCAGTACATCAATGGGCG

*shRNA interference sequence inserts are marked in red font, where the AgeI digestion site is disrupted.

**Plasmid Extraction**

The correctly sequenced bacteriophage was transferred to 150 ml of LB liquid medium containing Amp antibiotics and incubated overnight at 37°C in shaking bed. Extract the plasmids according to the instructions of EndoFree Maxi Plasmid Kit, and the plasmids that pass the quality control will enter the downstream process.

Detailed operation steps are as follows.

1. Centrifuge at 8000rpm for 4min to collect the bacteriophage.

2. add 7ml of P1 and shake and mix.

3. add 7ml P3, mix upside down 6~8 times and leave for 5min.

4. add 7ml P4, mix upside down 6~8 times, ice bath for 10min.

5. centrifuge at 9000rpm for 10min, transfer the supernatant to filter CS, filter and add 10ml of isopropanol and mix.

6. add 2.5ml of equilibrium solution BL to the adsorption column, centrifuge at 8000rpm for 2min, pour off the waste solution in the collection tube, and put the column back into reserve.

7. pour the supernatant into the adsorption column in two portions, centrifuge at 8000 rpm for 2 min, and discard the waste solution.

8. add 10 ml of rinsing solution PW (anhydrous ethanol has been added) to the adsorption column, centrifuge at the same speed for 2 min, discard the waste solution, and repeat the step once again.

10. add 3ml of anhydrous ethanol to the adsorption column, centrifuge at 8000rpm for 2min and discard the waste solution.

11. shake in vacuo at 9500rpm for 5min to remove the residual rinse solution.

Transfer the adsorption column to a new white tube, add 800μl of elution buffer TB dropwise to the center of the column (preheat it first), leave it at room temperature for 5min, then centrifuge it at 9500rpm for 2min.

12. transfer the eluate from the tube to a clean 1.5 ml EP tube and store at -20°C.

13. Take samples for electrophoresis, determine the plasmid concentration using a spectrophotometer (Thermo_Nanodrop 2000), and quality check.

14. Transfer the QC-qualified plasmids to the downstream platform for virus packaging.
