# Supplementary figures and images for: FLVCR1 Predicts Poor Prognosis and Promotes Malignant Phenotype in Esophageal Squamous Cell Carcinoma via Upregulating CSE1L
Source: Front Oncol. 2021 Mar 25;11:660955. doi: 10.3389/fonc.2021.660955 (PMC8027484; doi:10.3389/fonc.2021.660955)

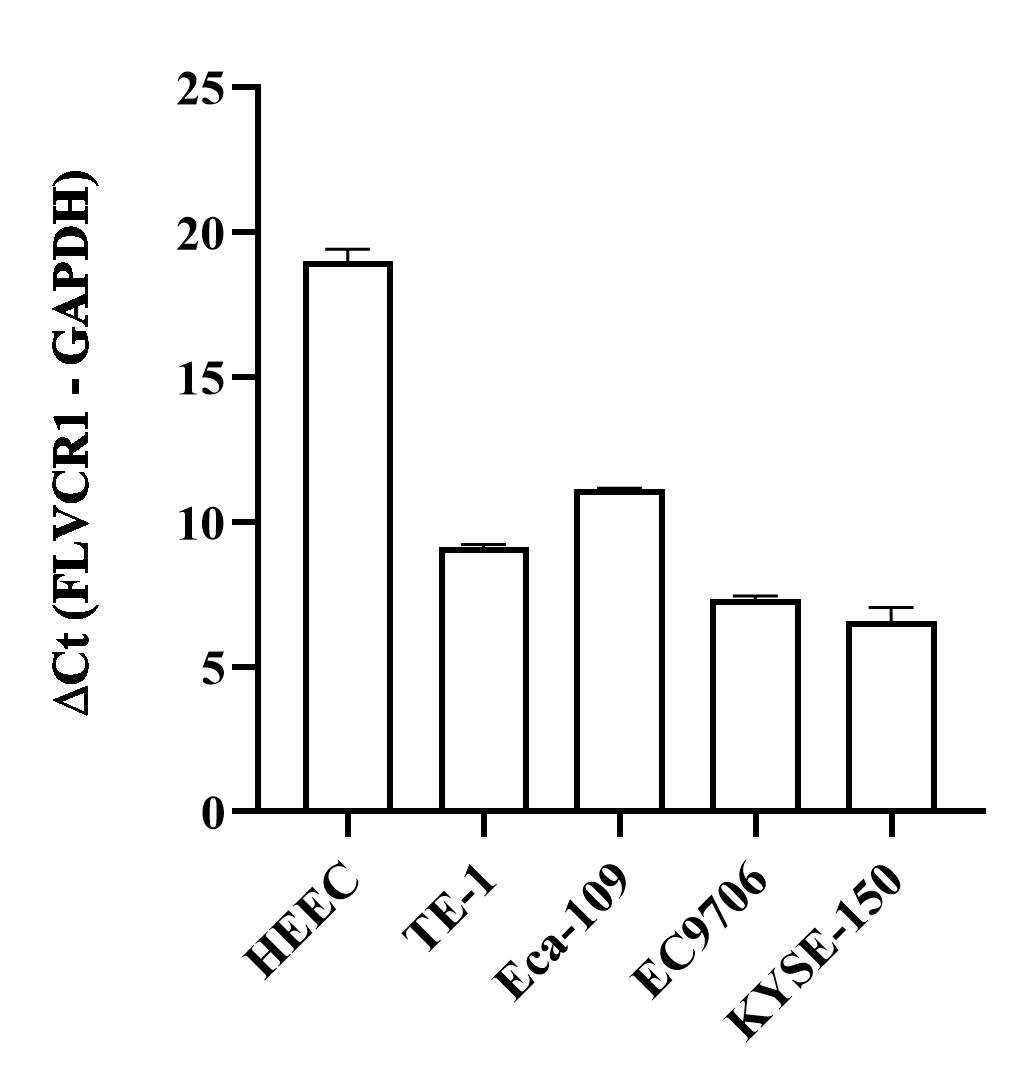

Supplement: Supplementary file 4 [file Image_1.tif]

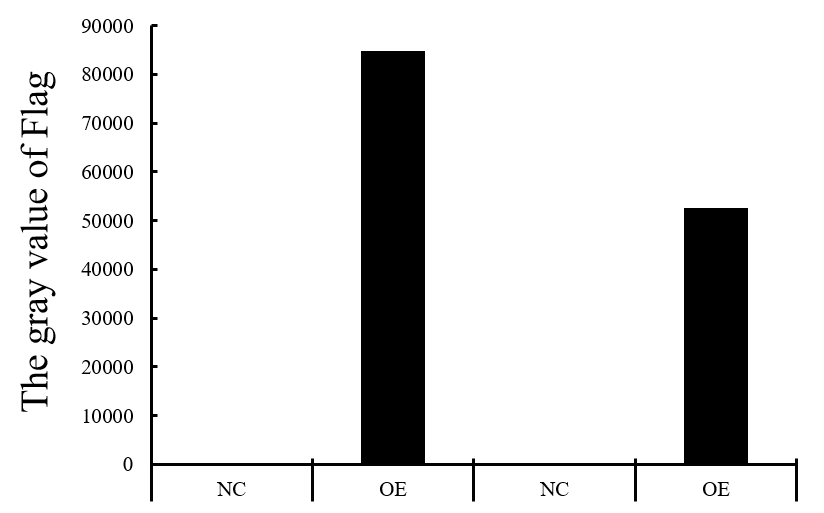

Supplement: Supplementary file 5 [file Image_2.tif]
